# An Autism-Associated Neuroligin-3 Mutation Affects Developmental Synapse Elimination in the Cerebellum

**DOI:** 10.3389/fncir.2021.676891

**Published:** 2021-06-28

**Authors:** Esther Suk King Lai, Hisako Nakayama, Taisuke Miyazaki, Takanobu Nakazawa, Katsuhiko Tabuchi, Kouichi Hashimoto, Masahiko Watanabe, Masanobu Kano

**Affiliations:** ^1^Department of Neurophysiology, Graduate School of Medicine, The University of Tokyo, Tokyo, Japan; ^2^Department of Physiology, Division of Neurophysiology, School of Medicine, Tokyo Women's Medical University, Tokyo, Japan; ^3^Department of Anatomy, Hokkaido University Graduate School of Medicine, Sapporo, Japan; ^4^Department of Functioning and Disability, Faculty of Health Sciences, Hokkaido University, Sapporo, Japan; ^5^Department of Bioscience, Tokyo University of Agriculture, Tokyo, Japan; ^6^Department of Molecular and Cellular Physiology, Shinshu University School of Medicine, Matsumoto, Japan; ^7^Department of Neurophysiology, Graduate School of Biomedical and Health Sciences, Hiroshima University, Hiroshima, Japan; ^8^International Research Center for Neurointelligence (IRCN), The University of Tokyo Institutes for Advanced Study, The University of Tokyo, Tokyo, Japan

**Keywords:** synapse elimination, neuroligin-3 mutation, autism, developing cerebellum, climbing fibers, Purkinje cell, mouse

## Abstract

Neuroligin is a postsynaptic cell-adhesion molecule that is involved in synapse formation and maturation by interacting with presynaptic neurexin. Mutations in neuroligin genes, including the arginine to cystein substitution at the 451st amino acid residue (R451C) of neuroligin-3 (NLGN3), have been identified in patients with autism spectrum disorder (ASD). Functional magnetic resonance imaging and examination of post-mortem brain in ASD patients implicate alteration of cerebellar morphology and Purkinje cell (PC) loss. In the present study, we examined possible association between the R451C mutation in NLGN3 and synaptic development and function in the mouse cerebellum. In NLGN3-R451C mutant mice, the expression of NLGN3 protein in the cerebellum was reduced to about 10% of the level of wild-type mice. Elimination of redundant climbing fiber (CF) to PC synapses was impaired from postnatal day 10–15 (P10–15) in NLGN3-R451C mutant mice, but majority of PCs became mono-innervated as in wild-type mice after P16. In NLGN3-R451C mutant mice, selective strengthening of a single CF relative to the other CFs in each PC was impaired from P16, which persisted into juvenile stage. Furthermore, the inhibition to excitation (I/E) balance of synaptic inputs to PCs was elevated, and calcium transients in the soma induced by strong and weak CF inputs were reduced in NLGN3-R451C mutant mice. These results suggest that a single point mutation in NLGN3 significantly influences the synapse development and refinement in cerebellar circuitry, which might be related to the pathogenesis of ASD.

## Introduction

Establishment of proper neural circuits relies on dynamic processes of synapse formation and elimination/pruning. Supernumerary synapses are formed transiently around birth, yielding excessive excitatory synaptic connections. Subsequently during postnatal development, some synapses are strengthened, whereas others are weakened and eventually eliminated in a neural activity-dependent manner (Purves and Lichtman, 1980; Lichtman and Colman, 2000; Hua and Smith, 2004; Kano and Hashimoto, 2009). Accumulating evidence strongly suggests that abnormality in developmental synapse elimination underlies the pathophysiology of neurodevelopmental and psychiatric disorders including autism spectrum disorder (ASD) (Zoghbi, 2003; Penzes et al., 2011). ASD is a category of pervasive developmental disorder characterized by impaired social interaction or communication, and stereotyped or repetitive behaviors (Geschwind and Levitt, 2007; Fombonne, 2009). ASD is highly hereditary, numerous ASD-associated genes have been identified, and a number of ASD mouse models have been reported (Abrahams and Geschwind, 2008; Bourgeron et al., 2009; Tsai et al., 2012a). Majority of ASD-associated genes are thought to encode synaptic proteins such as synaptic cell adhesion molecules, neuroligin and neurexin families (Südhof, 2008; Singh and Eroglu, 2013; Maćkowiak et al., 2014; Stewart, 2015) and a scaffold protein in the postsynaptic density, SHANK/ProSAP (Berkel et al., 2010; Arons et al., 2012; Guilmatre et al., 2014).

Previous studies show that some autism-related genes are involved in synapse elimination/pruning. For instance, myocyte enhancer factor 2 (MEF2) and Fragile X mental retardation 1 (Fmr1) induce excitatory synapse elimination in mouse hippocampal neurons. Deletion of these genes resulted in excessive dendritic spine formation (Pfeiffer et al., 2010; Tsai et al., 2012a). Moreover, mutations in genes that act to inhibit mammalian target of rapamycin (mTOR) kinase, including Tsc1/Tsc2, NF1, and Pten are reported to increase the dendritic spine density with reduced spine pruning in layer V pyramidal neurons of the temporal lobe of postmortem ASD patients and in cortical projection neurons of Tsc2-deficient mice (Tang et al., 2014). However, it remains unclear how impairment of synapse elimination/pruning contributes to the pathophysiology of ASD.

Neuroligins are postsynaptic cell-adhesion molecules that are comprised of four isoforms and are involved in synapse formation and maintenance by interacting with presynaptic neurexin. While neuroligin-1 (NLGN1), neuroligin-2 (NLGN2), and neuroligin-4 (NLGN4) are specifically expressed in excitatory, inhibitory and glycinergic synapses, respectively; neuroligin-3 (NLGN3) is present at both excitatory and inhibitory synapses (Chih et al., 2005; Varoqueaux et al., 2006; Craig and Kang, 2007; Bolliger et al., 2008). Single gene mutation in the arginine to cysteine substitution at the 451st amino acid (R451C) of NLGN3 has been identified in several ASD patients (Jamain et al., 2003; Yan et al., 2005). The R451C point mutation in mice shows a deviation of the balance between excitatory and inhibitory synaptic inputs from that of wild-type mice in the hippocampus and somatosensory cortex (Tabuchi et al., 2007; Etherton et al., 2011). However, it is unclear whether the R451C point mutation of NLGN3 affects synapse elimination/pruning in the developing brain.

The cerebellum has been suggested to be associated with the pathophysiology of ASD by studies on human patients and various animal models. Live imaging studies in ASD patients showed cerebellar abnormalities including hypoplasia of the vermis, gray and white matter abnormalities and cerebellar undergrowth, which are present in early life and persist into adulthood (Courchesne et al., 1988; Becker et al., 2001; Palmen and van Engeland, 2004; DiCicco-Bloom et al., 2006; Webb et al., 2009; Aldinger et al., 2013; Sundberg and Sahin, 2015). Postmortem studies in ASD patients showed loss of Purkinje cells (PCs), excessive number of Bergmann glia, or activation of microglia and production of cytokine in the cerebellar white matter (Bailey et al., 1998; Kern, 2003; Bauman and Kemper, 2005; Vargas et al., 2005; Sundberg and Sahin, 2015). Moreover, studies on ASD-related animal models revealed reduced number of PCs, dysfunction of PCs and deficits in cerebellum-dependent associative learning (Shahbazian et al., 2001; Tsai et al., 2012b; Reith et al., 2013; Kloth et al., 2015; Peter et al., 2016; Hoxha et al., 2017; Xiao et al., 2020; Yamashiro et al., 2020). These studies raise a further question whether and how altered development of synaptic wiring in the cerebellum is related to ASD.

To address these issues, we examined whether climbing fiber (CF) to PC synapse elimination in the developing cerebellum, a representative model of developmental synapse elimination, is affected in mice with R451C point mutation of NLGN3 (NLGN3-R451C mutant mice) (Tabuchi et al., 2007). In the neonatal mouse cerebellum, each PC receives excitatory synaptic inputs on the soma from multiple (more than five) CFs that originate from neurons in the inferior olive of the medulla. The strengths of multiple CF synaptic inputs are similar around birth, but inputs from a single CF selectively become stronger relative to those from the other CFs in each PC during the first postnatal week. Then, only the strongest CF extends its synaptic territory along the growing PC dendrite. In parallel, synapses from weaker CFs are eliminated from the soma, and most PCs become innervated by single strong CFs on their proximal dendrites by the end of the third postnatal week (Watanabe and Kano, 2011; Hashimoto and Kano, 2013; Kano et al., 2018; Kano and Watanabe, 2019). We found a marked reduction of NLGN3 protein expression in the cerebellum and enhanced synaptic inhibition of PCs leading to an elevation of the inhibition to excitation balance of synaptic inputs to PCs in NLGN3-R451C mutant mice. CF to PC synapse elimination was impaired transiently from postnatal day 10–15 (P10–P15), but CF innervation pattern became normal after P16. Furthermore, selective strengthening of a single CF relative to the other weaker CFs in each PC was impaired from P16 to juvenile stage because the weaker CFs remained abnormally strong. We assume that these changes during CF to PC synapse development leave persistent effects on the operation of cerebellar neural circuits, which might contribute to the ASD-like behavioral abnormalities in NLGN3-R451C mutant mice.

## Materials and Methods

### Animals

All experiments were performed in accordance with the guidelines for the care and use of laboratory animals of the University of Tokyo and the Japan Neuroscience Society. NLGN3-R451C mutant mice were generated on the 129/SvJ background and have been backcrossed to the C57BL/6 strain for more than 10 generations (Tabuchi et al., 2007). Litters of mice were kept in a room at 22°C with 12 h dark-light cycles and were weaned at P21. Weaned pups were housed in the same-sex group of 4–6. Standard rodent pellets and water were provided *ad libitum*. In total, 77 male wild-type and 74 male homozygote NLGN3-R451C mice were used in the present study. All experiments in the present study were performed under the conditions in which the experimenters were blind to the mouse genotypes.

### Electrophysiology and Ca^2+^ Imaging

Mice were deeply anesthetized with CO_2_ and killed by decapitation. The brain was quickly removed and placed in the chilled (0–4°C) artificial cerebrospinal fluid (ACSF) containing (in mM) 125 NaCl, 2.5 KCl, 2 CaCl_2_, 1 MgSO_4_, 1.25 NaH_2_PO_4_, 26 NaHCO_3_, and 20 glucose, bubbled with 95% O_2_ and 5% CO_2_ (pH 7.4). Parasagittal cerebellar slices (250 μm thick) were prepared from the cerebellar vermis of mice aged P4–P35 using a vibratome slicer (VT-1200S, Leica, Germany). Whole-cell recordings were made from visually identified somata of PCs at 32°C as described previously (Hashimoto et al., 2009b) using an upright microscope (BX50WI, Olympus, Japan). We used three intracellular solutions with the following compositions (in mM): (1) 60 CsCl, 10 D-gluconate, 20 TEA-Cl, 20 BAPTA, 4 MgCl_2_, 4 ATP, 0.4 GTP, and 30 HEPES (pH 7.3, adjusted with CsOH) for recording excitatory postsynaptic currents (EPSCs), (2) 124 CsCl, 10 HEPES, 10 BAPTA, 1 CaCl_2_, 4.6 MgCl_2_, 4 ATP, 0.4 GTP (pH 7.3, adjusted with CsOH) for recording inhibitory postsynaptic currents (IPSCs), and (3) 135 K-gluconate, 10 Na-gluconate, 5 KCl, 0.5 EGTA, 10 HEPES, 4 Mg-ATP, 0.4 Na_3_-GTP (pH 7.3, adjusted with NaOH), and 0.1 Oregon Green 488 BAPTA1 for recording CF-induced complex spikes and resultant calcium transients. Picrotoxin (100 μM) was added to block GABA receptor-mediated inhibitory currents for recording EPSCs, and NBQX (10 μM) and D-AP5 (50 μM) were included to block AMPA receptor-mediated and NMDA receptor-mediated excitatory synaptic currents, respectively, for recording IPSCs. To stimulate CFs in the granular layer underneath the recorded PCs, electrical pulses (duration of 0.1 ms, amplitude of 0–100 V) were applied at 0.2 Hz through a glass pipette filled with the normal ACSF (Hashimoto and Kano, 2003; Hashimoto et al., 2009b). CF-mediated EPSCs (CF-EPSCs) were recorded at a holding potential of −10 mV to avoid inadvertent generation of voltage-dependent regenerative responses (Hashimoto and Kano, 2003; Hashimoto et al., 2009b). The number of CFs innervating the recorded PC was estimated according to the number of discrete CF-EPSC steps as previously described (Hashimoto and Kano, 2003; Hashimoto et al., 2009b). To search all CFs innervating the recorded PC, we moved the stimulation pipette systematically around the PC soma and increased the stimulus intensity gradually at each stimulation site (Nakayama et al., 2012; Kawata et al., 2014). To measure the inhibitory/excitatory (I/E) ratio, PFs were stimulated in the molecular layer at the position where a maximum response was elicited with the stimulus current of 5 μA. D-AP5 was present throughout the experiment to block NMDA receptors. AMPA receptor-mediated currents evoked by PF-stimulation were first recorded at a holding potential of −60 mV, the reversal potential for GABA receptor-mediated current under our experimental conditions. PCs were subsequently depolarized to −20 mV, NBQX was applied to block AMPARs, and GABA_A_ receptor-mediated synaptic currents were recorded. The I/E ratio was calculated by dividing the peak GABA_A_ receptor-mediated current by the peak AMPA receptor-mediated current.

For Ca^2+^-imaging, Oregon Green 488 BAPTA-1 (0.1 mM; Molecular Probes) was included in the internal solution and applied to the recorded PC by diffusion for at least 20 min. Fluorescence images were acquired by using a high-speed confocal laser scanning microscope (CSU22, Yokogawa, Japan) before and after the application of a 1 s depolarization pulse from −70 to 0 mV to the recorded PCs, as described previously (Tanimura et al., 2009). At each time point, the Ca^2+^-dependent fluorescence signals from selected regions of interest (ROIs) in the soma and dendrite were corrected by subtracting the background fluorescence signal from a ROI outside the recorded PC. The Ca^2+^-dependent fluorescence signal was expressed as an increase in fluorescence divided by the pre-stimulus fluorescence values (ΔF/F0) using the Image J software (http://rsbweb.nih.gov/ij/). The changes in fluorescence signals induced by CF inputs in PC somatic or dendritic regions were recorded (Nakayama et al., 2012).

### Quantification of Disparity in Multiple CF-EPSCs

To quantify the disparity in multiple CF-EPSCs of a given PC, we calculated disparity ratio, a parameter which reflects the average of the inverse proportion of the strongest CF-EPSC amplitude to each of the other weaker CF-EPSCs (Hashimoto and Kano, 2003). For calculating the disparity ratio, the amplitude of individual CF-EPSCs in a given multiply innervated PC were measured and they were numbered in the order of their amplitudes (A_1_, A_2_,…, A_N_, N ≧ 2, A_N_ represents the largest CF-EPSC). The disparity ratio was obtained from the following formula.

$$\begin{array}{l} \text{Disparity ratio}=\left({\text{A}}_{1}/{\text{A}}_{\text{N}}+{\text{A}}_{2}/{\text{A}}_{\text{N}}+\ldots \ldots +{\text{A}}_{\text{N}-1}/{\text{A}}_{\text{N}}\right)/\\ \;\left(\text{N}-1\right) \end{array}$$

### Western Blot Analysis

Proteins from the cerebella of 2-month-old NLGN3-R451C mutant mice and wild-type mice were extracted and homogenized in RIPA buffer (125 mM Tris-HCl, ph 6.8; 10% Mercaptoethanol, 0.004% Bromophenol Blue, 10% Sucrose, and 4% SDS) with a pestle and mortar. Protein concentrations were determined using the Bradford assay (Bio-Rad Laboratories). Equal amounts of proteins were loaded to the 10% polyacrylamide gel and run at 196 V and 40 mA for 90 min. The blot was performed at 25 V and 100 mA for 2 h. The primary antibodies were applied overnight at 4°C and HRP-conjugated secondary antibodies were incubated for 1 h at room temperature. The development was performed with Western Lightning Plus ECL (PerkinElmer) and ImageQuant LAS 4000 (GE Healthcare) was used to visualize the bands.

### Immunohistochemistry

All primary antibodies were purchased from Nittobo Medical Co., Ltd. (Tokyo, Japan). Under deep pentobarbital anesthesia (100 μg/g of body weight, i.p.), wild-type and NLGN3-R451C mutant mice with 30–35 days of age were fixed with ice-cold 4% paraformaldehyde in 0.1 M sodium phosphate buffer (pH 7.4) and processed for preparation of parasagittal cerebellar sections (50 μm thickness). Free-floating sections were incubated overnight with affinity-purified primary antibodies against the following molecules (host species, final concentration, RRID): NLGN3 (rabbit, 1μg/ml, AB_2571813), anti-calbindin (goat, 1μg/ml, AB_2571569), VGluT2 (rabbit, 1 μg/ml, AB_2619683), parvalbumin (guinea pig, 1 μg/ml, AB_2571615), and vesicular inhibitory amino acid transporter (VIAAT) (rabbit, 0.5 μg/ml, AB_2571622). Sections were incubated subsequently with a mixture of species-specific secondary antibodies labeled with Alexa 488 (1:200, Invitrogen, Carlsbad, CA), Cy3, and Cy5 (1:200, Jackson ImmunoResearch, West Grove, PA) for 2 h. Slices were then washed and mounted on glass slides with Vectashield mounting media (Vector Laboratories). Images were taken with confocal laser-scanning microscope (LSM510, Zeiss). Quantitative analyses of the number of PCs, the length of primary PC dendrite and the number of molecular layer interneurons (**Figures 5O–Q**) were performed on lobule 3, 4/5, and 6 of the cerebellar slices by using MetaMorph software (Molecular Devices, Sunnyvale, CA).

### Anterograde Tracer Labeling

Three wild-type and three NLGN3-R451C mutant mice were used for the following analyses. Under anesthesia by inhalation of 3.5% isoflurane, a glass pipette filled with 2–3 μl of 10% solution of dextran Alexa Fluor-594 (DA-594, Invitrogen) in PBS was inserted stereotaxically into the inferior olive by the dorsal approach, as described previously (Miyazaki and Watanabe, 2011). The tracer was injected by air pressure (Pneumatic Picopump; World Precision Instruments). After 4 days of the tracer injection, mice were deeply anesthetized with sodium pentobarbital (100 μg/g of body weight, i.p.) and fixed by transcardial perfusion at P14 or P28. To visualize tracer-labeled CFs, all CF terminals, and PC somata and dendrites, microslicer sections of the cerebellum from DA-594-injected mice were incubated overnight with a mixture of primary antibodies against calbindin (a marker for PC; rabbit serum, 1:10,000 dilution; AB_2571568) and VGluT2 (a marker for CF terminals; goat, 1 μg/ml, AB_2571620), followed by 2 h incubation with a mixture of species-specific secondary antibodies as described above. Images of the triple labeling were taken with a confocal laser-scanning microscope (FV1000, Olympus, Tokyo, Japan).

### Post-embedding Immunogold Electronmicroscopy

For postembedding immunogold, three NLGN3-R451C mutant and three wild-type mice with 30–35 days of age were used. Cerebellar sections (250 μm in thickness) were embedded in Lowicryl HM20 (Lowi) medium and polymerized with ultraviolet. The ultrathin sections processed with an ultramicrotome (ULTRACUT UCT, Lecia) were incubated overnight with rabbit anti-NLGN3 antibody (5 μg/ml) and then with 10-nm colloidal gold-conjugated anti-rabbit IgG (1:100; British Bio Cell International). Subsequently, the sections were incubated overnight with guinea pig anti-VGluT1 (10 μg/ml), anti-VGluT2 (10 μg/ml), and anti-VIAAT (10 μg/ml) antibodies and then with 15-nm colloidal gold-conjugated anti-guinea pig IgG (1:100; British Bio Cell International). Electron micrographs were taken with an JEM1400 electron microscope (JEOL, Tokyo, Japan). For the quantitative analysis of synaptic localization of immune-gold particles (**Figure 4G**), we used MetaMorph software (Molecular Devices, Sunnyvale, CA).

### Statistics

Data are presented as mean ± SD unless indicated otherwise. Unpaired two-tailed Student's *t*-test, Mann-Whitney U-test or the Kolmogorov-Smirnov test was used for comparison of two independent samples. Two-way ANOVA and the non-parametric Kruskal-Wallis H test were used for multiple comparisons. Statistical analysis was conducted with GraphPad Prism 8 software (GraphPad Software, La Jolla, CA, USA) and IBM SPSS statistics (IBM SPSS Inc., Chicago, IL, USA). *p* < 0.05 was considered as statistically significant.

## Results

### Selective Strengthening of Single CF Inputs Relative to Others Is Compromised in NLGN3-R451C Mutant Mice

**Figure 1:**
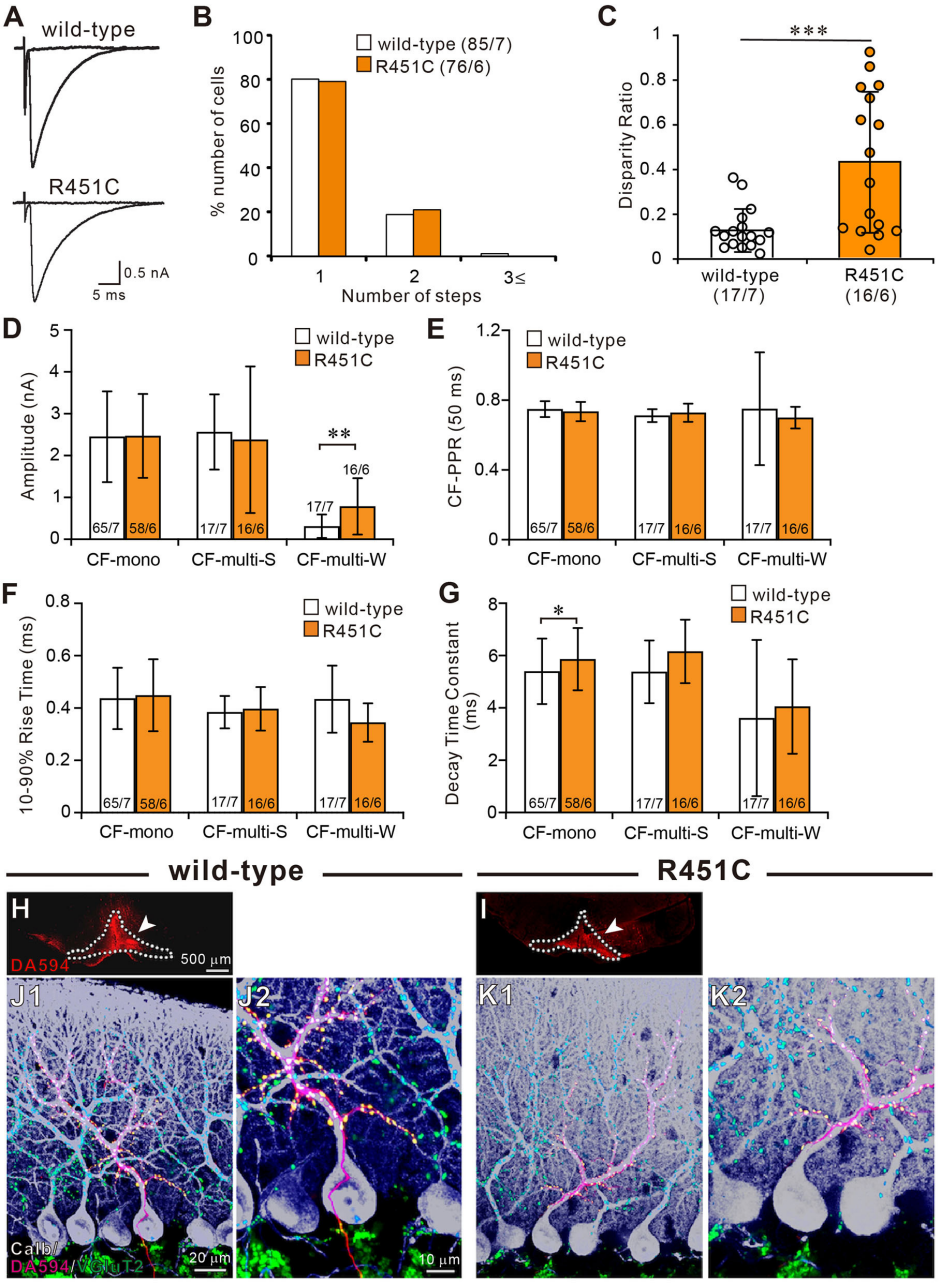
The NLGN3-R451C mutation impairs functional differentiation of multiple CF inputs during synapse elimination. **(A)** Specimen traces of CF-EPSC recorded from Purkinje cell soma in wild-type and NLGN3-R451C mutant (R451C) mice at P21–P35 at a holding potential (Vh) of −10 mV in the presence of 100 μM picrotoxin. Scale bars, 5 ms and 0.5 nA. **(B)** Frequency distribution of the number of CF-EPSC steps in each PCs for wild-type (open columns) and NLGN3-R451C mutant (orange columns) mice at P21–P35. *p* = 0.9 by Mann-Whitney U test. Numbers of PCs/mice are shown in parentheses. **(C)** Summary plots of disparity ratio in wild-type (open circles and open column) and NLGN3-R451C mutant (orange circles and orange column) mice during P21–P35. ***p < 0.001 by Mann-Whitney U test. Numbers of PCs/mice are shown in parentheses below the graph. Data are expressed as mean ± SD. **(D–G)** Summary bar graphs for the amplitude **(D)**, paired-pulse ratio **(E)**, 10–90% rise time **(F)**, and decay time constant **(G)** of EPSCs elicited by stimulating CF-mono, CF-multi-S, and CF-multi-W in wild-type (open column) and NLGN3-R451C mutant (orange column) mice. Data were obtained from P21–P35 mice and are expressed as mean ± SD. **p = 0.0061 in **(D)** and *p = 0.033 in **(G)** by Mann-Whitney U test. Numbers of PCs/mice are shown inside or above the bar. **(H,I)** Fluorescent images of the anterograde tracer (red, DA-594) showing its injection sites (arrows) in the inferior olive (dotted lines) of wild-type **(H)** and NLGN3-R451C mutant **(I)** mice. **(J,K)** Triple immunostaining for calbindin (white), anterogradely labeled CFs (red, DA-594), and VGluT2 (green) in wild-type **(J1,J2)** and NLGN3-R451C mutant **(K1,K2)** mice at P28. **(J2,K2)** are the magnified views of **(J1,K1)**, respectively. Scale bars represent 500 μm for **(D,E)**, 20 μm for **(J1,K1)**, and 10 μm for **(J2,K2)**. Three mice at P28 of each genotype were used for **(H–K)**.

To determine CF innervation patterns of PCs in juvenile wild-type and mutant mice, we performed whole-cell recordings from PCs in cerebellar slices from mice aged P21–P35. In 80% (68/85) of PCs in wild-type mice and 79% (60/76) in NLGN3-R451C mutant mice, single large CF-EPSCs were induced in an all-or-none fashion as the stimulus intensity was gradually increased (Figure 1A). There was no significant difference in the frequency distribution of PCs in terms of the number of CF-EPSC steps between wild-type and NLGN3-R451C mutant mice (Figure 1B). This result suggests that CF elimination is completed normally in PCs of juvenile NLGN3-R451C mutant mice.

We then examined whether the selective strengthening of a single CF among multiple CFs in each PC was affected in NLGN3-R451C mutant mice. We calculated the disparity ratio for each multiply innervated PC (see section Materials and Methods), which reflects the average of the inverse proportion of the strongest CF-EPSC amplitude to each of the other weaker CF-EPSCs (Hashimoto and Kano, 2003). We found that the disparity ratio was significantly larger in juvenile NLGN3-R451C mutant mice than in wild-type mice (Figure 1C), suggesting that selective strengthening of a single CF in each PC is severely impaired in NLGN3-R451C mutant mice. Since each PC is either mono innervated by a single strong CF (CF-mono) or multiply innervated by a strong CF (CF-multi-S) and a few weaker CFs (CF-multi-W), we compared the amplitude of EPSCs for these three categories of CFs (Figure 1D). Whereas the amplitudes of EPSCs for CF-mono and for CF-multi-S were similar between the two genotypes, those for CF-multi-W were significantly larger in NLGN3-R451C mutant mice than in wild-type mice (Figure 1D), which appeared to be the basis for the elevated disparity ratio in NLGN3-R451C mutant mice. In contrast, basic electrophysiological properties of EPSCs for the three categories of CFs were similar between the two genotypes (Figures 1E–G) except that the decay time constant for CF-mono was longer in NLGN3-R451C mutant mice (Figure 1G).

Next, we examined the innervation pattern of CFs morphologically by labeling a subset of CFs with an anterograde tracer, DA-594, injected into the inferior olive (Figures 1H,I). Combining with immunofluorescence for a PC marker, calbindin and a global CF terminal marker, VGluT2, we found that DA-594-labeled CFs precisely followed the PC's proximal dendrites and climbed up to the four-fifths of the molecular layer in both wild-type and NLGN3-R451C mutant mice (Figures 1J,K). VGluT2 signals were only found on proximal dendrites but not in the somatic regions of PCs, which indicates essentially all the VGluT2 signals overlapped with DA-594 signals. This result demonstrates that most of the wild-type and NLGN3-R451C mutant PCs are innervated by single CFs on their proximal dendrites. There was no appreciable difference between the two genotypes.

### CF Synapse Elimination Is Delayed From P10 to P15 in NLGN3-R451C Mutant Mice

**Figure 2:**
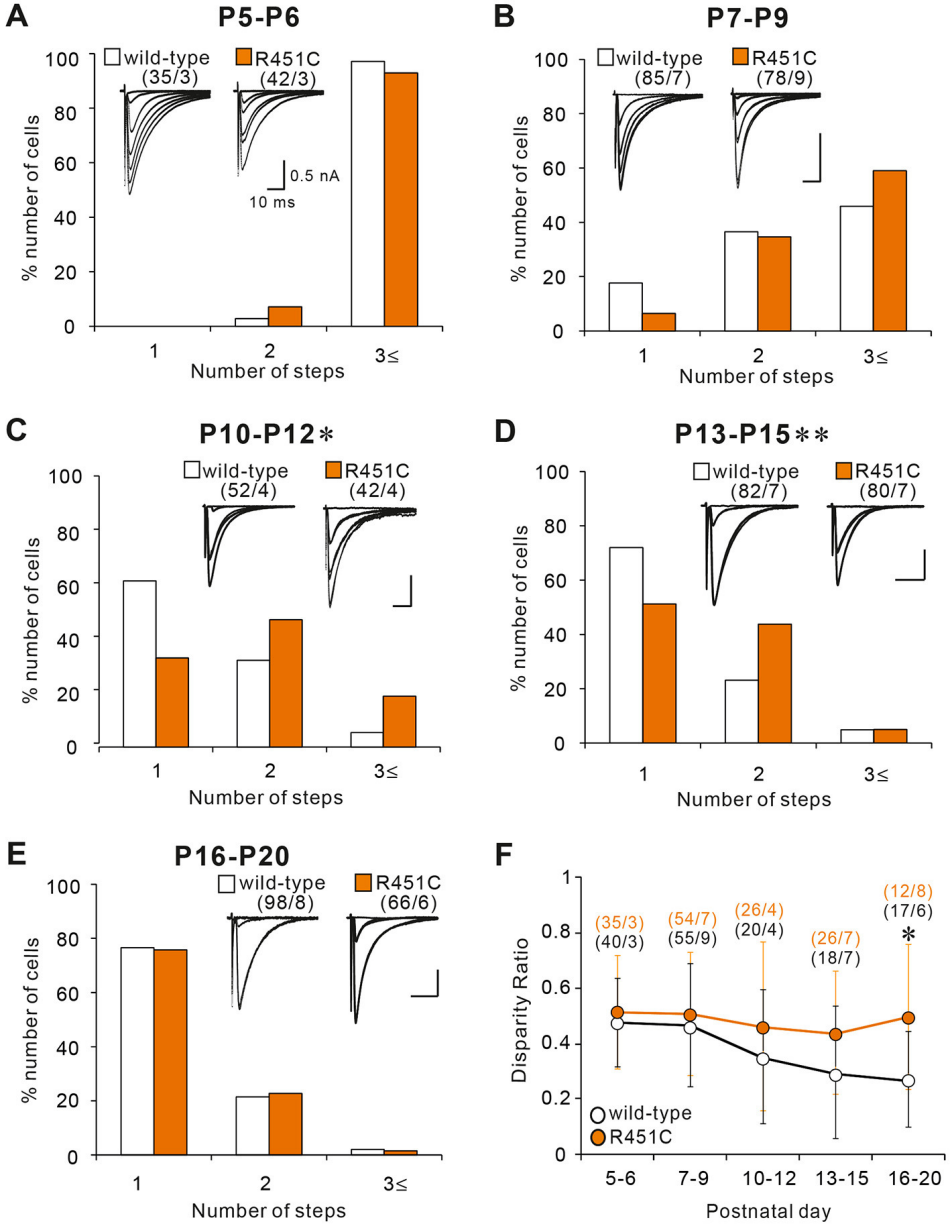
The NLGN3-R451C mutation impairs CF synapse elimination during postnatal development. **(A–E)** Specimen records of CF-EPSCs (inserts, five to eight traces were superimposed at each threshold intensity; Vh = −10 mV) and frequency distribution histogram for the number of discrete CF-EPSCs for wild-type (open columns) and NLGN3-R451C mutant mice (orange columns) at indicated ages. There is no significant difference in the frequency distribution in **(A,B,E)** between wild-type and NLGN3-R451C mutant mice [**(A)**, *p* = 0.143, **(B)**, *p* = 0.063, and **(E)**, *p* = 0.6 by Mann-Whitney U test]. In contrast, frequency distributions for **(C,D)** are significantly different between wild-type and NLGN3-R451C mutant mice (**C**, *p = 0.024 and **D**, **p < 0.001 by Mann-Whitney U test). Scale bars, 10 ms and 0.5 nA. Numbers of PCs/mice are shown in parentheses. **(F)** Summary plots of disparity ratio in wild-type (open circles) and NLGN3-R451C mutant (orange circles) mice at indicated ages. There is a significant difference between wild-type and NLGN3-R451C mutant mice aged P16–P20 (P5–P6, *p* = 0.961; P7–P9, *p* = 0.877; P10–P12, *p* = 0.409; P13–P15, *p* = 0.173; P16–P20; *p = 0.039 by Two-way ANOVA with Tukey's *post-hoc* test). Numbers of PCs/mice are shown in parentheses. Data are expressed as mean ± SD.

Although majority of PCs in juvenile NLGN3-R451C mutant mice were innervated by single CFs, functional differentiation of multiple CFs during postnatal development appeared to be affected. We therefore investigated whether CF innervation patterns during the first three postnatal weeks are altered in NLGN3-R451C mutant mice. From P5 to P9, majority of PCs were multiply innervated by CFs in both wild-type and NLGN3-R451C mutant mice. Although the number of CFs innervating individual PCs decreased from P5–P6 to P7–P9, no significant differences were found between the genotypes (Figures 2A,B). In contrast, a significantly higher number of CFs innervated individual PCs in NLGN3-R451C mutant mice than in wild-type mice from P10 to P12 (Figure 2C) and from P13 to P15 (Figure 2D). Then, the CF innervation pattern in NLGN3-R451C mutant mice became identical to that of wild-type from P16 to P20 (Figure 2E), which persisted into juvenile stage (Figure 1). We also calculated the disparity ratio at each of the five developmental stages (Figure 2F) and found that the disparity ratio in NLGN3-R451C mutant mice became larger than that in wild-type mice after P16. To check whether there is any structural change transiently in the developing cerebellum, we examined the innervation pattern of CFs morphologically at P14 by labeling a subset of CFs with DA-594 (Supplementary Figure 1). We found no perceptive difference between the genotypes in the morphology of PC dendrites and CF innervation pattern. These results indicate that regression of multiple CF innervation initially occurs normally until P9, is impaired transiently from P10 to P15, and then is resumed to yield normal pattern of CF innervation after P16. In contrast, impairment in the strengthening of a single CF relative to the other weaker CFs in each PC became obvious at around P16 and persisted thereafter.

### Reduced NLGN3 Expression but Normal Gross Anatomy and Cellular Morphology in the Cerebellum of NLGN3-R451C Mutant Mice

**Figure 3:**
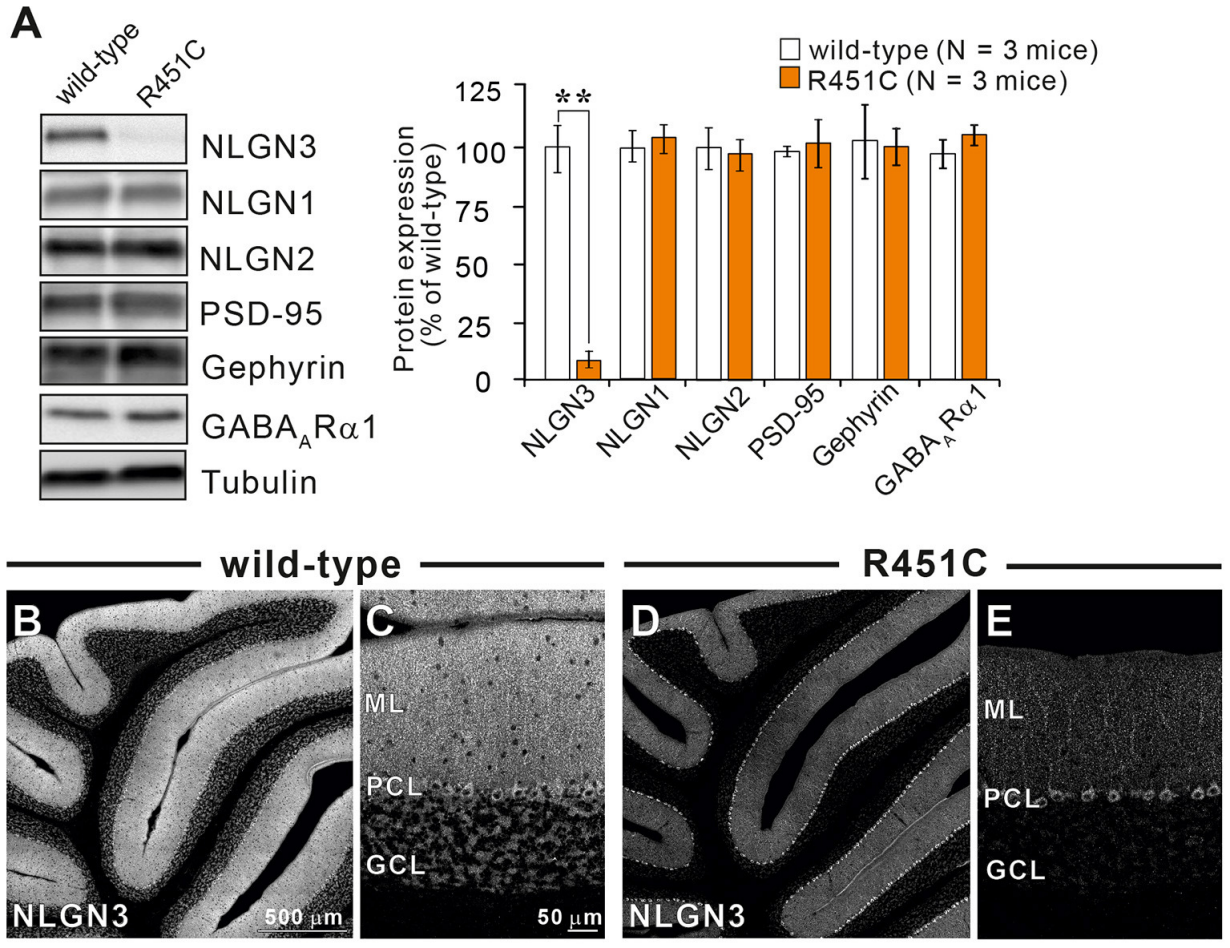
The NLGN3-R451C mutation greatly reduces the expression of NLGN3 but not other synaptic proteins in the cerebellum. **(A)** Representative immunoblots (left panel) and summary bar graphs (right panel) showing the expression levels of several synaptic proteins in cerebellar homogenates from 2-month-old wild-type (open columns) and age-matched NLGN3-R451C mutant mice (orange columns) (*N* = 3 mice/group). Representative synaptic proteins (NLGN1, neuroligin-1; NLGN2, neuroligin-2; NLGN3, neuroligin-3; PSD-95, Gephyrin, GABA_A_ receptor subunit α1) were analyzed by quantitative immunoblotting. Data are expressed as mean ± SD. **p < 0.001 by Student's *t*-test. **(B–E)** Immunohistochemistry for NLGN3 in the cerebellum with a low magnification **(B,D)** and with a high magnification **(C,E)** in 2-month-old wild-type **(B,C)** and age-matched NLGN3-R451C mutant **(D,E)** mice. ML, molecular layer; PCL, Purkinje cell layer; GCL, granule cell layer. In wild-type mice, NLGN3 signal is intense in ML and GL, whereas that in NLGN3-R451C mutant mice is decreased. Scales bars represent 500 μm for **(B,D)**, and 50 μm for **(C,E)**.

**Figure 4:**
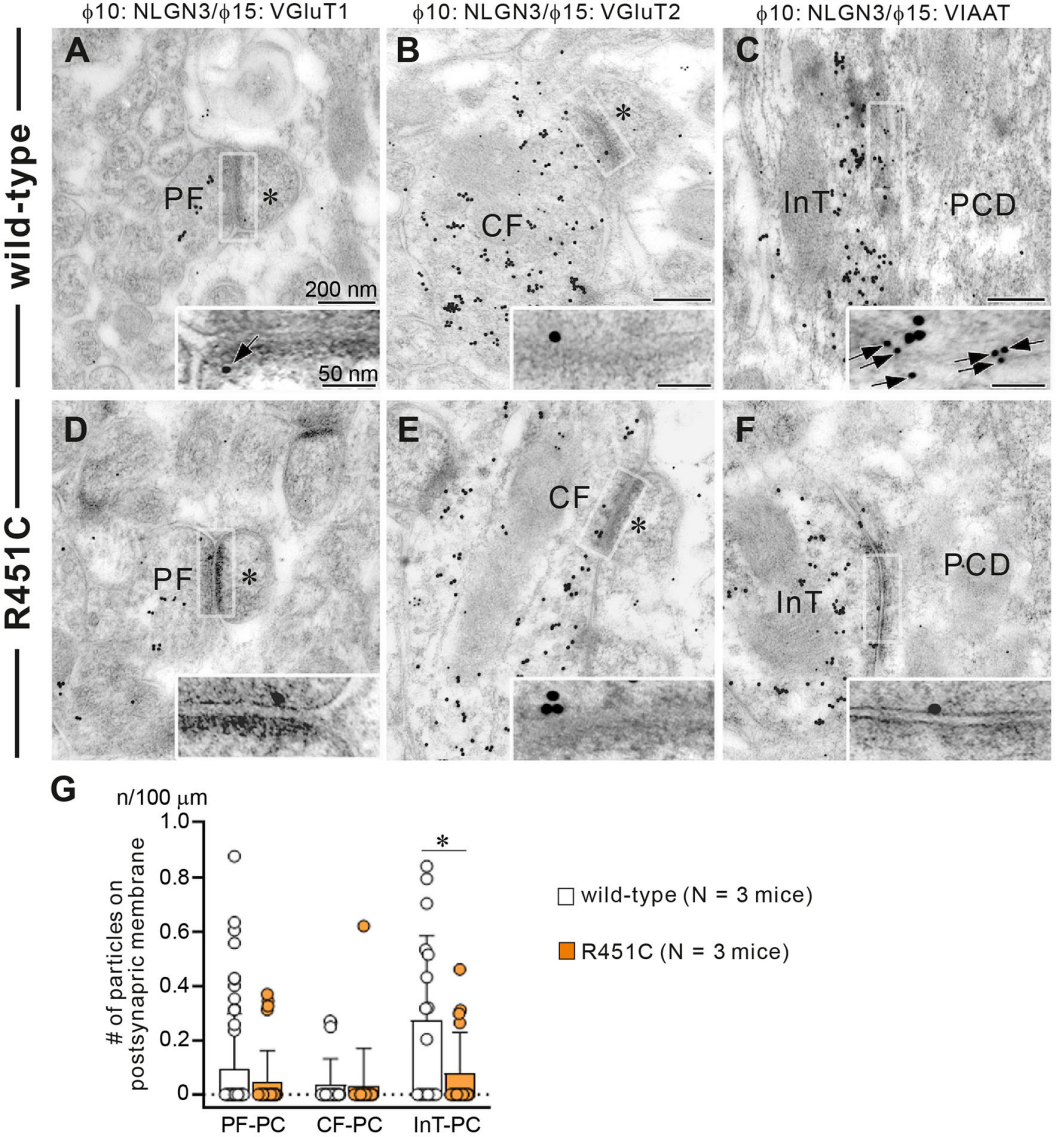
Reduced NLGN3 expression at inhibitory interneurons to PC synapses in NLGN3-R451C mutant mice. **(A–F)** Post-embedding immunogold electron microscopy for NLGN3 in wild-type **(A–C)** and NLGN3-R451C mutant **(D–F)** mice. **(A,D)** Indicate PC spines (asterisks) contacting to PF presynaptic terminals labeled for VGluT1. **(B,E)** Indicate PC spines (asterisks) contacting to CF presynaptic terminals labeled for VGluT2. **(C,F)** Indicate PC dendrites (PCD) contacting to presynaptic terminals of inhibitory interneurons (InT). Scale bars, 200 nm and 50 nm for inset. **(G)** Summary bar graphs showing the average number of NLGN3 immunogold particles (n) per 100 μm of the postsynaptic membrane of PF-PC, CF-PC, and InT-PC synapses in wild-type (open circles and columns, *N* = 3 mice aged 30–35 days) and NLGN3-R451C mutant (orange circles and columns, *N* = 3 mice aged 30–35 days) mice. Data are expressed as mean ± SD. *p* = 0.33 for PF-PC, *p* = 0.61 for CF-PC and *p = 0.035 for InT-PC synapses by Mann-Whitney U test.

**Figure 5:**
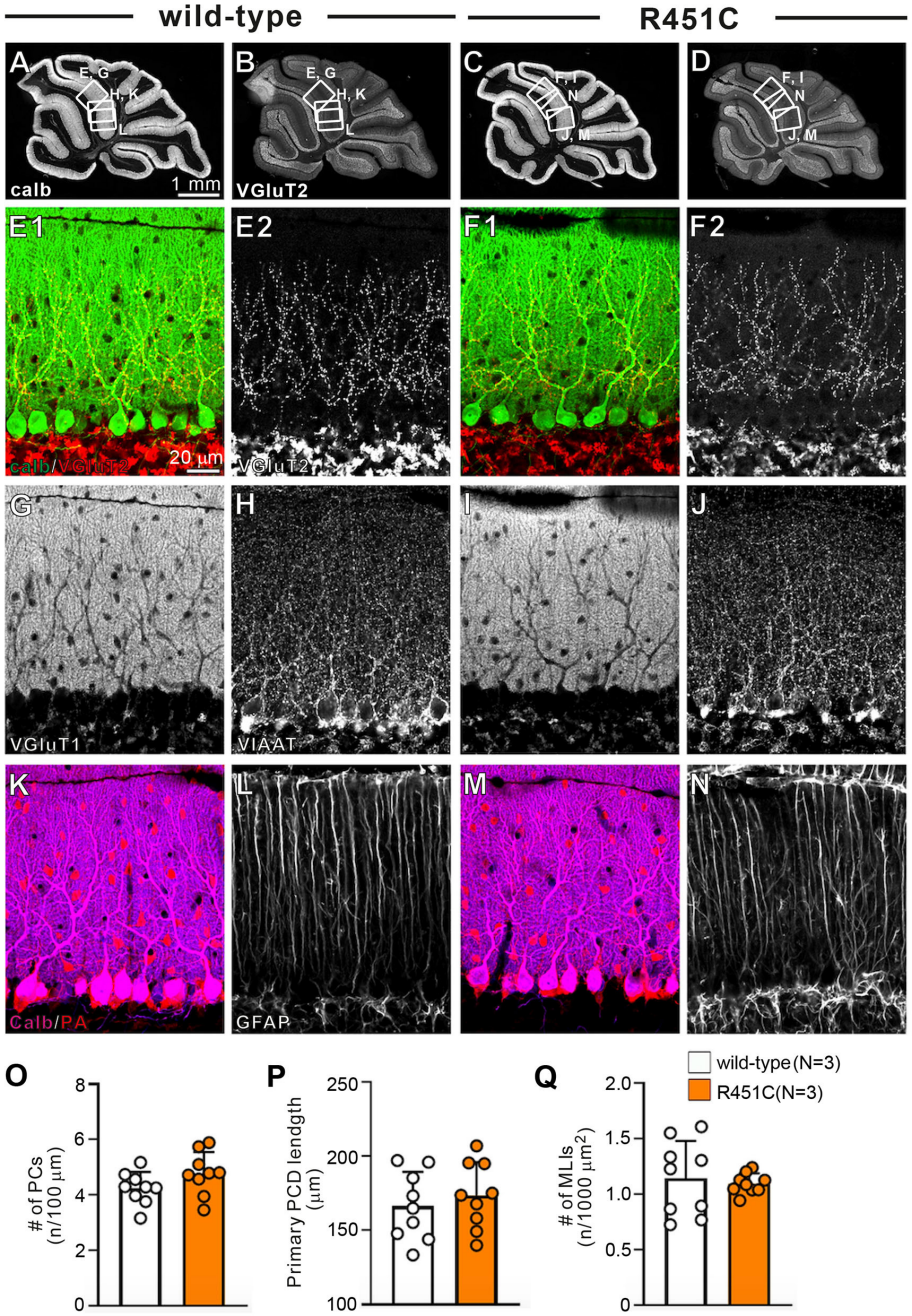
Gross anatomy of the cerebellum, morphology of major cell types, and density of major excitatory and inhibitory synapses appear normal in NLGN3-R451C mutant mice. **(A–D)** Immunofluorescence for the PC marker calbindin **(A,C)** and for the CF terminal marker VGluT2 **(B,D)** of the cerebellum of wild-type **(A,B)** and NLGN3-R451C mutant **(C,D)** mice. The boxed regions in **(A,B)** and those in **(C,D)** indicate the portions of the cerebellum from which the images in **(E,G,H,K,L)** and those in **(F,I,J,M,N)** were obtained, respectively. Scale bar, 1 mm. **(E–N)** Immunofluorescence for calbindin (green, **E1,F1**) and VGluT2 [red for **(E1,F1)**, white for **(E2,F2)**], for the PF marker VGluT1 **(G,I)**, for the inhibitory synaptic terminal marker vesicular inhibitory amino acid transporter (VIAAT) **(H,J)**, for a marker of PC and inhibitory interneurons, parvalbumin (PA) **(K,M)**, and for the astrocyte marker glial fibrillary acidic protein (GFAP) **(L,N)** in wild-type **(A,E,G,H,K,L)** and NLGN3-R451C mutant **(F,I,J,M,N)** mice. Scale bar, 20 μm. **(O–Q)** Histograms for wild-type (open circles and columns, *N* = 3 mice aged 30–35 days) and NLGN3-R451C mutant mice (orange circles and columns, *N* = 3 mice aged 30–35 days) showing the number of PCs (n) per 100 μm of Purkinje cell layer **(O)**, the length of primary PC dendrite **(P)** and the number of MLIs (n) per 1000 μm^2^ **(Q)**. Data were obtained from the straight portions of lobule 3, 4/5, and 6, and are expressed as mean ± SD. *p* = 0.11 for **(O)**, *p* = 0.55 for **(P)** and *p* = 0.80 for **(Q)** by Mann-Whitney *U* test.

In a previous work on the NLGN3-R451C mutation, Etherton and coworkers (Etherton et al., 2011) showed that NLGN3 controls excitatory and inhibitory synaptic transmission in circuit-dependent and brain region-specific manners. For instance, mutant mice displayed an increase in synaptic inhibition in the somatosensory cortex while an increase in AMPA receptor-mediated excitatory synaptic transmission and NMDA receptor containing GluN2B subunits in the hippocampus. Given NLGN3 is localized to both glutamatergic and GABAergic synapses, organizes the scaffolding protein recruitment and maintains the synapse stability, we explored a possibility that the NLGN3-R451C mutation delays the CF to PC synapse elimination process by changing the expression level of synaptic molecules. We first analyzed the expression of synaptic proteins in the cerebellum. Consistent with the previous studies on other brain regions, we found that the NLGN3-R451C substitution caused a nearly 90% reduction of NLGN3 total protein (Figure 3A). However, we did not observe any significant changes in the expression of other NLGN isoforms (NLGN1 and NLGN2), PSD-95 (a marker of excitatory synapses), Gephyrin (a marker of inhibitory synapses) and GABA_A_Rα1 (GABA_A_ receptor subunit α1) (Figure 3A). To further characterize possible effects of the R451C mutation, we checked the cellular expression of NLGN3 within the cerebellum. Immunohistochemical study using an NLGN3-specific antibody showed that NLGN3 was strongly localized in the molecular layer, granular layer and PC layer in wild-type mice (Figures 3B,C), which is similar to a previous study (Baudouin et al., 2012). However, in NLGN3-R451C mutant mice, NLGN3 expression was decreased in both the molecular and the granular layers (Figures 3D,E). Post-embedding immunogold electron microscopy showed that the density of metal particles for NLGN3 at the postsynaptic membrane was not different between wild-type and NLGN3-R451C mutant mice for parallel fiber (PF) to PC synapses (Figures 4A,D,G) and for CF to PC synapses (Figures 4B,E,G). In contrast, the postsynaptic NLGN3 density was significantly lower in NLGN3-R451C mutant than in wild-type mice at inhibitory interneuron (InT) to PC synapses (Figures 4C,F,G). We also checked global histo- and cytoarchitecture of the cerebellum. No apparent difference between genotypes were observed in the size and lobular organization of the cerebellum (Figures 5A–D), the cellular arrangement and dendritic arborization of PCs (Figures 5E1,F1), the distribution and density of CF terminals (Figures 5E2,F2), PF terminals (Figures 5G,I), inhibitory terminals (Figures 5H,J), and cell bodies of molecular layer interneurons (Figures 5K,M), and the palisade-like arrangement of Bergmann glia's fibers (Figures 5L,N). No difference was found in the number of PCs (Figure 5O), the length of primary PC dendrite (Figure 5P) or the number of molecular layer interneurons (MLIs) (Figure 5Q).

### Elevated Inhibition to Excitation Balance in PCs of NLGN3-R451C Mutant Mice

**Figure 6:**
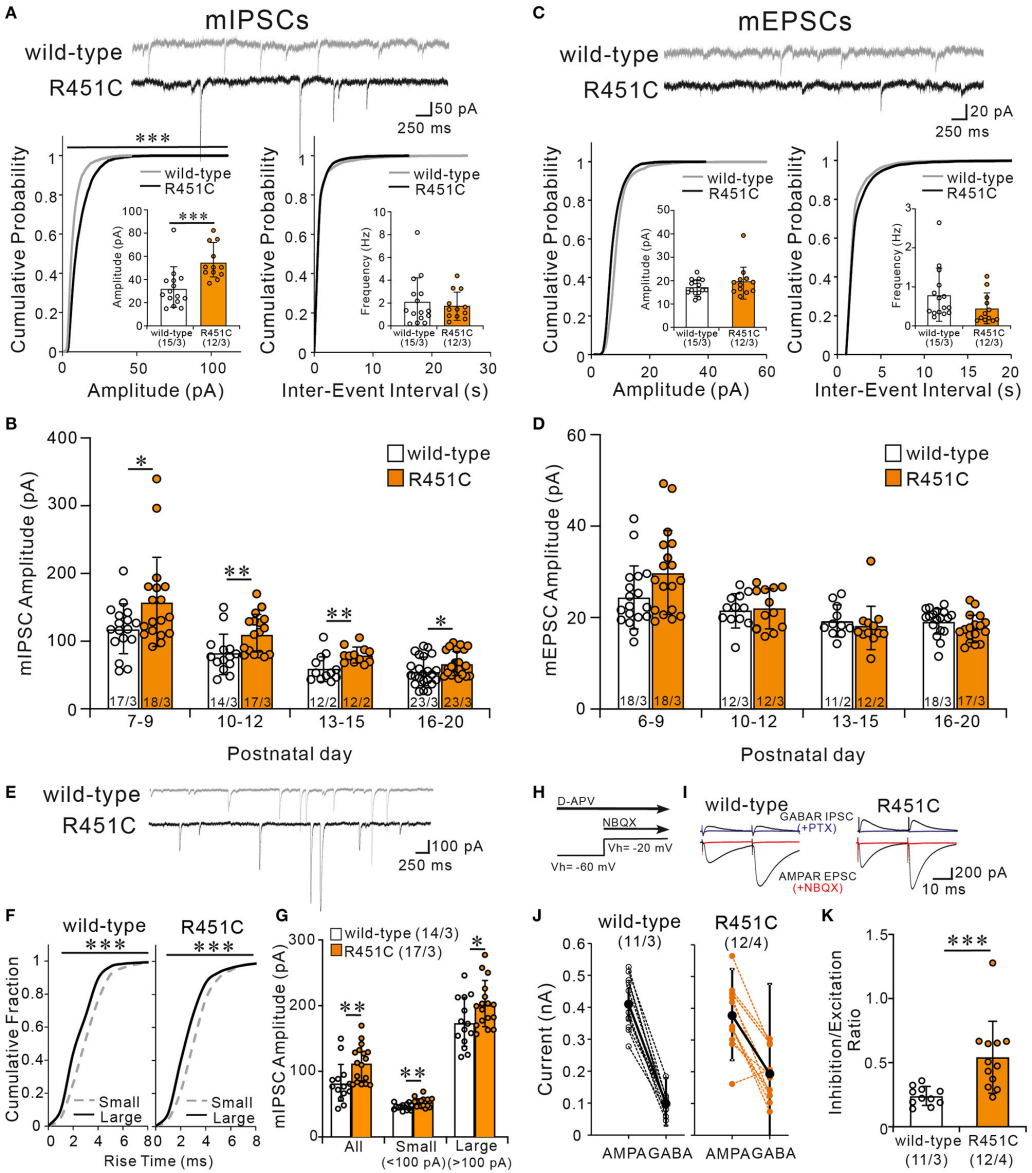
The NLGN3-R451C mutation causes the enhancement of inhibitory synaptic transmission and the elevation of inhibition/excitation ratio of synaptic inputs to PCs. **(A)** Representative traces of mIPSC recorded from PCs of wild-type (upper trace) and NLGN3-R451C mutant (lower trace) mice (top) at Vh of −70 mV in the presence of 1 μM tetrodotoxin, 10 μM NBQX, and 5 μM R-CPP (*N* = 3/group). Scale bars, 250 ms and 50 pA. Cumulative distribution plots and summary bar graphs for the mIPSC amplitude (lower left; inset shows the average mIPSC amplitude) and for the inter-event interval (lower right; inset shows the average mIPSC frequency) in PCs of wild-type and NLGN3-R451C mutant mice aged P21–P35. For the mIPSC amplitude, ***p < 0.0001 by Kolmogorov-Smirnov test in the cumulative distribution plot and ***p = 0.0002 by Mann-Whitney U test in the bar graph. For the inter-event interval, *p* = 0.99 by Kolmogorov-Smirnov test in the cumulative distribution plot and *p* = 0.9 by Mann-Whitney U test in the bar graph. **(B)** Summary graph for the mIPSC amplitude of wild-type (open columns) and NLGN3-R451C mutant (orange columns) mice at indicated ages. Note that the mean amplitudes of mIPSCs in NLGN3-R451C mutant mice are significantly larger than those of wild-type mice during postnatal development (P7–P9, *p = 0.029; P10–P12, **p = 0.0053; P13–P15, **p = 0.0083; P16–P20, *p = 0.024 by Mann-Whitney U test). **(C)** Representative traces of mEPSC recorded from PCs of wild-type (upper trace) and NLGN3-R451C mutant (lower trace) mice (top) at Vh of −70 mV in the presence of 1 μM tetrodotoxin and 100 μM PTX (*N* = 3/group). Scale bars, 250 ms and 20 pA. Cumulative distribution plots for the mEPSC amplitude (lower left; inset shows the average mEPSC amplitude) and for the inter-event interval (lower right; inset shows the average mEPSC frequency) in PCs of wild-type and NLGN3-R451C mutant mice aged P21–P35. For the mEPSC amplitude, *p* = 0.17 by Kolmogorov-Smirnov test in the cumulative distribution plot and *p* = 0.40 by Mann-Whitney U test in the bar graph. For the inter-event interval, *p* = 0.84 by Kolmogorov-Smirnov test in the cumulative distribution plot and *p* = 0.59 by Mann-Whitney U test in the bar graph. **(D)** Summary graph for the mEPSC amplitude of wild-type (open columns) and NLGN3-R451C mutant (orange columns) mice at indicated ages (P6–P9, *p* = 0.074; P10–P12, *p* = 0.76; P13–P15, *p* = 0.350; P16–P20, *p* = 0.10 by Mann-Whitney U test). **(E)** Representative traces of mIPSC recorded from PCs of wild-type (upper) and NLGN3-R451C mutant (lower) mice at P10 in the presence of 1 μM tetrodotoxin, 10 μM NBQX, and 5 μM R-CPP. Vh = −70 mV. Scale bars, 250 ms and 100 pA. **(F)** Cumulative fractions of the rise time of small (<100 pA) (gray dotted line) and large (>100 pA) (black line) mIPSCs in wild-type (left) and NLGN3-R451C mutant (right) mice from P10 to P12. ***p < 0.0001 by Kolmogorov-Smirnov test for both genotypes. **(G)** Summary bar graphs for the mIPSC amplitude for all, small and large events in wild-type (open columns) and NLGN3-R451C mutant (orange columns) mice from P10 to P12. **p = 0.0053 (All); **p = 0.0014 (Small); *p = 0.0484 (Large) by Mann-Whitney U test. **(H)** Experimental protocol used to measure the inhibition/excitation ratio. **(I)** Representative traces of evoked AMPAR- and GABA_A_R-mediated synaptic currents in wild-type (left) and NLGN3-R451C mutant (right) mice. Scale bars, 10 ms and 200 pA. **(J)** Amplitudes of AMPAR- (left) and GABA_A_R- (right) mediated synaptic currents from wild-type (black) and NLGN3-R451C mutant (orange) mice. **(K)** Average inhibition/excitation ratio from wild-type (open column) and NLGN3-R451C mutant (orange column) mice aged P19–P25. ***p = 0.0004 by Mann-Whitney U test. Data are expressed as mean ± SD. Total number of cells recorded/total number of mice used is indicated within or beneath individual columns.

How does the NLGN3-R451C mutation delays CF synapse elimination and compromises the strengthening of a single CF relative to the other weaker CFs? Because post-embedding immunogold electron microscopy showed a significant reduction of NLGN3 expression at inhibitory synapses of PC, we hypothesized that alteration in inhibitory synaptic strength to PCs may have caused the changes in CF to PC synapse development in NLGN-R451C mutant mice. We first examined miniature inhibitory postsynaptic currents (mIPSCs) from PCs of mice aged P22 to P35 and found that the amplitude but not the frequency was increased by ~50% in NLGN3-R451C mutant mice when compared to wild-type mice (Figure 6A). We then examined mIPSCs during postnatal development of CF to PC synapses. We found that the amplitude of mIPSCs was consistently larger in NLGN3-R451C mutant mice than in wild-type mice during P7–P9, P10–P12, P13–P15, and P16–P20 (Figure 6B). Our previous study shows that inhibitory synaptic inputs to PCs from around P10 to P12 are important for CF synapse elimination (Nakayama et al., 2012). We therefore scrutinized mIPSCs at this developmental stage and found that the amplitude of mIPSCs larger than 100 pA, which presumably arose from basket cells, and that smaller than 100 pA, which presumably represent inhibitory inputs from stellate cells, were both increased in NLGN3-R451C mutant mice compared to wild-type mice (Figures 6E–G).

In contrast to mIPSC, no significant differences were found in either the amplitude or the frequency of miniature excitatory postsynaptic currents (mEPSCs) between wild-type and NLGN3-R451C mutant mice aged P22–P35 (Figure 6C). Furthermore, the amplitude of mEPSCs was similar between genotypes throughout postnatal development (Figure 6D). We further examined EPSCs evoked by PF stimulation (PF-EPSCs) and found that the input-output relation and the response to paired stimulation (paired-pulse ratio) for PF-EPSCs were not altered in NLGN3-R451C mutant mice (Supplementary Figures 2A,B). Moreover, the paired-pulse ratio for CF-EPSCs was also not altered in NLGN3-R451C mutant mice (Supplementary Figures 2C,D).

Many previous studies indicate that the deviation of balance between inhibition and excitation in individual neurons underlies the pathophysiology of ASD (Tabuchi et al., 2007; Chao et al., 2010; Delorme et al., 2013). We therefore directly estimated the ratio of inhibition to excitation in individual PCs in response to stimulation in the molecular layer (Figures 6H–K). We first recorded PF-EPSCs that are mediated by AMPA receptors at a holding potential of −60 mV, which is equivalent to the Cl^−^ equilibrium potential, in the presence of the NMDA receptor antagonist D-AP5. Then we added NBQX to completely block AMPA receptor-mediated PF-EPSCs and recorded GABA_A_ receptor-mediated IPSCs at a holding potential of −20 mV, which were abolished by addition of PTX (Figures 6H,I). We then calculated the ratio of the amplitude of PF-EPSC to that of IPSC (I/E ratio) in each PC and found that the I/E ratio was markedly increased in NLGN3-R451C mutant mice (Figures 6J,K). Taken together, these results indicate that inhibitory synaptic inputs to PCs are enhanced but the overall strength of excitatory synaptic inputs to PCs are not altered, leading to elevated I/E balance in NLGN3-R451C mutant mice when compared to wild-type mice.

### Decreased CF-Induced Ca^2+^ Transients in the PC Soma in NLGN3-R451C Mutant Mice

**Figure 7:**
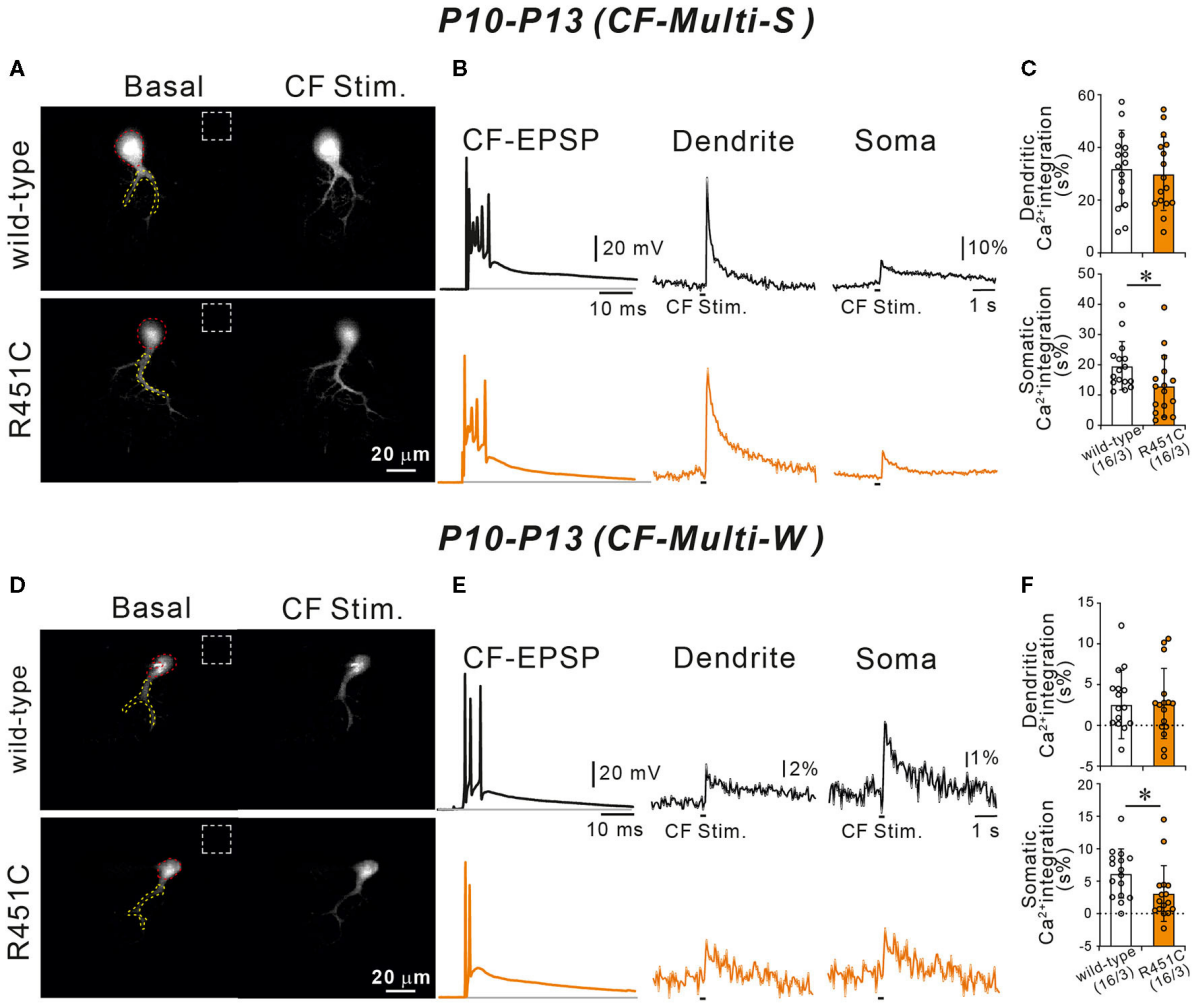
Ca^2+^ transients induced by strong and weak CF inputs are smaller in NLGN3-R451C mutant mice than in wild-type mice. **(A)** Representative images of a wild-type (upper left) and a NLGN3-R451C mutant (lower left) PC. Areas demarcated by red and yellow dotted lines represent ROIs for somatic and dendritic Ca^2+^ transients, respectively. Dashed rectangles represent the ROIs where the background fluorescence signals were measured. Scale bar, 20 μm. **(B)** Representative traces of CF-induced EPSP (right) and those of Ca^2+^ transients recorded in the dendrite (middle) and the soma (right) of multiply-innervated PCs in response to stimulation of the strongest CF in wild-type (black) and NLGN3-R451C mutant (orange) mice. Scale bars, 20 mV and 10 ms for CF-EPSC, 10% and 1 s for Ca^2+^ transients. **(C)** Average magnitudes of Ca^2+^ transients from the soma and the dendrite by stimulating the strongest CF in wild-type (open columns) and NLGN3-R451C mutant (orange columns) mice. *p* = 0.77 (Dendrite) and *p = 0.026 (Soma) by Mann-Whitney U test. **(D)** Representative images of a wild-type (upper left) and a NLGN3-R451C mutant (lower left) PC shown similarly to **(A)**. Scale bar, 20 μm. **(E,F)** Representative traces **(E)** and average magnitudes of Ca^2+^ transients **(F)** shown similarly to **(A,B)**, respectively, but for responses to stimulation of a weak CF of a multiply innervated PC. Scale bars, 20 mV and 10 ms for CF-EPSC, 2% or 1% and 1 s for Ca^2+^ transients. *p* = 0.86 (Dendrite) and *p = 0.015 (Soma) by Mann-Whitney U test. Data for **(A–F)** were obtained from P10 to P13 mice. Data in **(C,F)** are expressed as mean ± SD. Numbers of PCs/mice are shown in parentheses.

We have reported previously that diminished GABAergic transmission from putative basket cell to PC resulted in larger Ca^2+^ transients elicited by the weaker CFs in multiply innervated PCs from P10 to P13 (Nakayama et al., 2012). Because the R451C substitution of NLGN3 increases the amplitude of mIPSCs in PCs, CF-induced Ca^2+^ transients may be affected. To test this possibility, we recorded postsynaptic membrane potentials and Ca^2+^ transients from multiply innervated PCs from P10 to P13 during stimulation of CF-multi-S or CF-multi-W under current-clamp mode in the normal external solution. Stimulation of CF-multi-S induced characteristic complex spikes accompanied by clear Ca^2+^ transients in proximal dendrites and somata in both wild-type and NLGN3-R451C mutant mice (Figures 7A,B). We quantified the magnitude of CF-induced Ca^2+^ transients by measuring the integral of Ca^2+^ signals (for 1.5 s from the onset). We found that the Ca^2+^ transients in the soma were significantly smaller but those in dendrites were similar in NLGN3-R451C mutant mice compared to wild-type mice (Figure 7C). Stimulation of CF-multi-W induced smaller EPSPs with a few action potentials and much smaller Ca^2+^ transients when compared to those induced by stimulating CF-multi-S (Figures 7D,E). Notably, dendritic Ca^2+^ transients were very small and sometimes undetectable, which is consistent with the fact that CF-multi-W does not undergo dendritic translocation but stays around the PC soma (Hashimoto and Kano, 2003; Hashimoto et al., 2009a). Nevertheless, dendritic Ca^2+^ transients were similar between the two genotypes. In contrast, somatic Ca^2+^ transients induced by CF-multi-W were significantly smaller in NLGN3-R451C mutant mice than in wild-type mice (Figure 7F). These results indicate that increased inhibition to PCs results in reduced somatic Ca^2+^ transients elicited by CF-multi-S and CF-multi-W in PCs of NLGN3-R451C mutant mice presumably because of weaker activation of P/Q-type voltage-dependent calcium channels (P/Q-VDCCs) and resultant reduction of Ca^2+^ influx into the PC soma.

## Discussion

Several previous studies suggest that abnormal synaptic pruning/elimination in the developing brain underlies the pathophysiology of ASD (Weiler and Greenough, 1999; Bourgeron, 2009; Tsai et al., 2012a). For instance, in Fragile X syndrome patients and Fmr1 knockout mice, the number of dendritic spines in mature cortical neurons was increased because of the deficit in MEF2-dependent synapse elimination (Irwin et al., 2001, 2002; Pfeiffer et al., 2010). In Tsc2 mutant mice, the density of dendritic spines in cortical neurons was elevated because of over-activation of mTOR signaling, leading to the reduction of autophagy for spine pruning (Bourgeron, 2009; Tang et al., 2014). However, it is not clear whether and how ASD-associated genes are involved in synapse pruning/elimination in the developing cerebellum. In the present study, we show that an autism-associated genetic mutation of NLGN3 affects developmental synaptic refinement in the mouse cerebellum. In NLGN3-R451C mutant mice, CF to PC synapse elimination was delayed transiently from P10 to P15. Although CF innervation pattern became normal in juvenile mice, the weaker CF synaptic inputs were abnormally strong in NLGN3-R451C mutant PCs, which became evident from P16 and persisted into juvenile stage. These results indicate that abnormalities during postnatal development may perturb proper formation of cerebellar neural circuits and may cause lifelong effects on animal's behavior. However, it should be noted that NLGN3-R451C mutation is present not only in PCs but also in all the cell types that express NLG3 in the mutant mice. It is therefore possible that the abnormality of CF to PC synapse development in the mutant mice may result from, or at least may be influenced by, NLGN3-R451C mutation in cells other than PCs such as neurons in the inferior olive. It would be interesting in future to examine whether a PC-specific introduction of NLGN3-R451C mutation in mice causes abnormalities seen in global NLGN3-R451C mutant mice.

We found that the R451C substitution of NLGN3 caused a marked reduction of NLGN3 expression within the cerebellum to about 10% of the level of wild-type mice, and that significant reduction was found at inhibitory interneuron to PC synapses. During the postnatal development in NLGN3-R451C mutant mice, inhibitory neurotransmission onto PCs was enhanced without a noticeable effect on excitatory transmission. We found that both large and small mIPSCs recorded from NLGN3-R451C mutant PCs, which presumably arise from basket cells and stellate cells, respectively, were increased and the I/E ratio was elevated significantly. We therefore assume that NLGN3 in the cerebellar circuitry is mainly responsible for the regulation of inhibitory interneuron to PC synaptic transmission, although NLGN3 is widely localized at glutamatergic and GABAergic synapses.

How does a reduction of NLGN3 expression leads to an enhancement of inhibitory synaptic transmission in PCs? Tabuchi et al. (2007) reported increased inhibitory but normal excitatory synaptic transmission in the somatosensory cortex of NLGN3-R451C mutant mice (Tabuchi et al., 2007). They found increased levels of an inhibitory presynaptic protein, VIAAT, and an inhibitory postsynaptic protein, gephyrin, increased density of VIAAT puncta, but normal number of inhibitory synapses. The frequency of mIPSC was elevated, the response to applied GABA was increased, but the amplitude of mIPSC and short-term plasticity were normal. These changes in inhibitory synapses are not consistent with those found in cerebellar PCs of NLGN3-R451C mutant mice. We found an increase in the amplitude but no change in the frequency of mIPSCs and apparently normal intensity of VIAAT immunostaining in the cerebellum of NLGN3-R451C mutant mice. These results suggest that NLGN3-R451C mutation alters inhibitory synaptic function in cell type- and brain region-specific manners, while the outcome of increased inhibitory synaptic transmission and elevated I/E balance are the same. Moreover, Foldy et al. (2013) reported an increase in inhibitory synaptic transmission from cholecystokinin (CCK) basket cells to pyramidal cells in the hippocampus of NLGN3 R451C mutant, which resulted from the absence of endocannabinoid-mediated tonic inhibition (Foldy et al., 2013). It remains to be investigated in future studies how NLGN3-R451C mutation in PCs enhances inhibitory synaptic transmission and whether endocannabinoid-mediated tonic inhibition is absent in PCs of NLGN3-R451C mutant mice.

As the involvement of inhibitory transmission in autism-related synapse pruning was unclear in the previous studies, the cerebellum of NLGN3-R451C mutant mice provides a good model to examine whether and how inhibitory transmission contributes to developmental synapse elimination in ASD. The enhanced inhibition of PCs is considered to be a cause of transient impairment of CF synapse elimination from P10 to P15. On the other hand, our previous study on GAD67^+/GFP^ mice showed that reduced GABAergic inhibition to PCs from P10 impairs CF synapse elimination (Nakayama et al., 2012). It is currently unknown why enhancement and reduction of PC inhibition resulted in apparently similar phenotypes. It is possible that proper balance of excitation and inhibition is crucial for proper CF synapse elimination and deviation of the I/E balance from the normal range may lead to its impairment (see below).

Another key finding in the present study is that the synaptic strength of weaker CFs of multiply innervated juvenile PCs was stronger in NLGN3-R451C mutant mice than in wild-type mice, albeit normal CF innervation of PCs in juvenile mice. In neonatal mice, PCs were innervated by multiple CFs with similar synaptic strengths. From P3 to P7, a single CF is selectively strengthened among the multiple CFs in each PC (functional differentiation) (Hashimoto and Kano, 2005; Hashimoto et al., 2009a; Watanabe and Kano, 2011). Then, only the strengthened CF (“winner” CF) extends its innervation along growing dendrites in each PC (CF translocation) from P9 (Hashimoto and Kano, 2005; Hashimoto et al., 2009a; Watanabe and Kano, 2011). In parallel, the other weaker CFs (“loser” CFs) remaining on the soma are eliminated from P7 to P11 (early phase of CF innervation, which is independent of PF to PC synapse formation) and from P12 to P17 (late phase of CF innervation, which requires normal PF to PC synapse formation) (Hashimoto and Kano, 2005; Hashimoto et al., 2009a; Watanabe and Kano, 2011). Our previous study on PC-selective P/Q-type VDCC knockout mice demonstrated that preferential strengthening of a single CF input from multiple CFs in each PC is severely impaired from P5 to P8. During this developmental period, Ca^2+^ transients induced by spontaneous CF inputs were smaller in PCs of knockout mice (Hashimoto et al., 2011). These results indicate that Ca^2+^ transients in PCs by activation of P/Q-type VDCC are crucial for selective strengthening of a single winner CF and suppressing the other loser CFs in each PC.

We hypothesize that activation of the strong CF may produce “punishment signals” that depress the other weaker CF inputs without affecting the strong CF itself. The hypothetical “punishment signals” are assumed to require large Ca^2+^ transients for their production and can depress only weak CF inputs that generate small Ca^2+^-transients. We also hypothesize the presence of “survival signals” that are produced by Ca^2+^ transients in PCs and are necessary for the maintenance of CF inputs. In NLGN3-R451C mutant mice, activation of the strong CF induced smaller Ca^2+^ transients in the soma than in wild-type mice, which may have not produced sufficient “punishment signals.” Therefore, the weaker CFs may have not been eliminated and CF synapse elimination was transiently impaired from P10 to P15 in NLGN3-R451C mutant mice. However, subsets of the weaker CFs in NLGN3-R451C mutant mice may have not been able to produce sufficient “survival signals” and therefore they were eventually eliminated. Thus, the weaker CF inputs of NLGN3-R451C mutant mice that survived into juvenile stage may be stronger than those of wild-type mice presumably because of the reduced production of the hypothetical punishment signals from the strongest CF inputs. It is demonstrated that semaphorin 3A (Uesaka et al., 2014), progranulin (Uesaka et al., 2018), and Bai3 (Kakegawa et al., 2015; Sigoillot et al., 2015) in PCs function as “maintenance factors” for CFs, but it is not known whether these molecules require Ca^2+^ transients in PCs for their action. It is also reported that Arc/Arg3.1 (Mikuni et al., 2013), semaphorin 7A (Uesaka et al., 2014), and BDNF (Choo et al., 2017) in PCs function as “punishment signals” for weak CFs. While Arc/Arg3.1 is activated by Ca^2+^ elevation in PCs through P/Q-VDCC (Mikuni et al., 2013), semaphorin 7A and BDNF function at the downstream of metabotropic glutamate receptor 1 but not P/Q-VDCC (Uesaka et al., 2014; Choo et al., 2017). It remains to be investigated whether and how these reported molecules are involved or yet unidentified molecules play roles in shaping CF to PC synaptic wiring during postnatal development.

Many studies indicate that defects in neural circuits including the cerebellum are critical for ASD (O'Halloran et al., 2012; Piochon et al., 2014; Wang et al., 2014; Kloth et al., 2015; Stoodley et al., 2017; Kelley et al., 2020). The cerebellum has connections not only with motor areas of the cerebral cortex but also with the association cortices responsible for higher brain functions (Schmahmann, 2019). Live imaging and postmortem studies in ASD patients showed abnormalities in the cerebellum (Palmen et al., 2004; Wegiel et al., 2010; Becker and Stoodley, 2013) and damage to the cerebellum in neonates is a high risk of ASD (Limperopoulos et al., 2014; Wang et al., 2014). In mouse models of ASD, the cerebellum is reported to be responsible for ASD-like behaviors. For example, PC-specific knockout of an ASD-associated gene, Tsc, is shown to exhibit behavioral abnormalities relevant to ASD including impaired sociality and enhanced repetitive behavior (Tsai et al., 2012b). The results of the present study suggest that the second postnatal week of CF to PC synapse elimination may be critical for shaping neural circuits involving the cerebellum and the cerebrum, and impairment of this process may contribute to ASD-like behavioral abnormalities in NLGN3 R451C mutant mice (Tabuchi et al., 2007). Moreover, NLGN3-R451C mutant mice have been reported to display a significantly increased performance in the accelerating rotarod test because of their elevated trait of repetitive behavior, which is thought to result from impaired neural circuit function in the striatum (Rothwell et al., 2014).

## Data Availability Statement

The raw data supporting the conclusions of this article will be made available by the authors.

## Ethics Statement

The animal study was reviewed and approved by the experimental animal ethics committees of the University of Tokyo.

## Author Contributions

EL and MK designed the project and wrote the manuscript. EL, HN, TM, and TN conducted experiments. EL, TM, and TN analyzed the data. KT provided the NLGN3-R451C mutant mice. All authors contributed to the article and approved the submitted version.

## Conflict of Interest

The authors declare that the research was conducted in the absence of any commercial or financial relationships that could be construed as a potential conflict of interest.
