# Supplementary figures and images for: An Autism-Associated Neuroligin-3 Mutation Affects Developmental Synapse Elimination in the Cerebellum
Source: Front Neural Circuits. 2021 Jun 28;15:676891. doi: 10.3389/fncir.2021.676891 (PMC8273702; doi:10.3389/fncir.2021.676891)

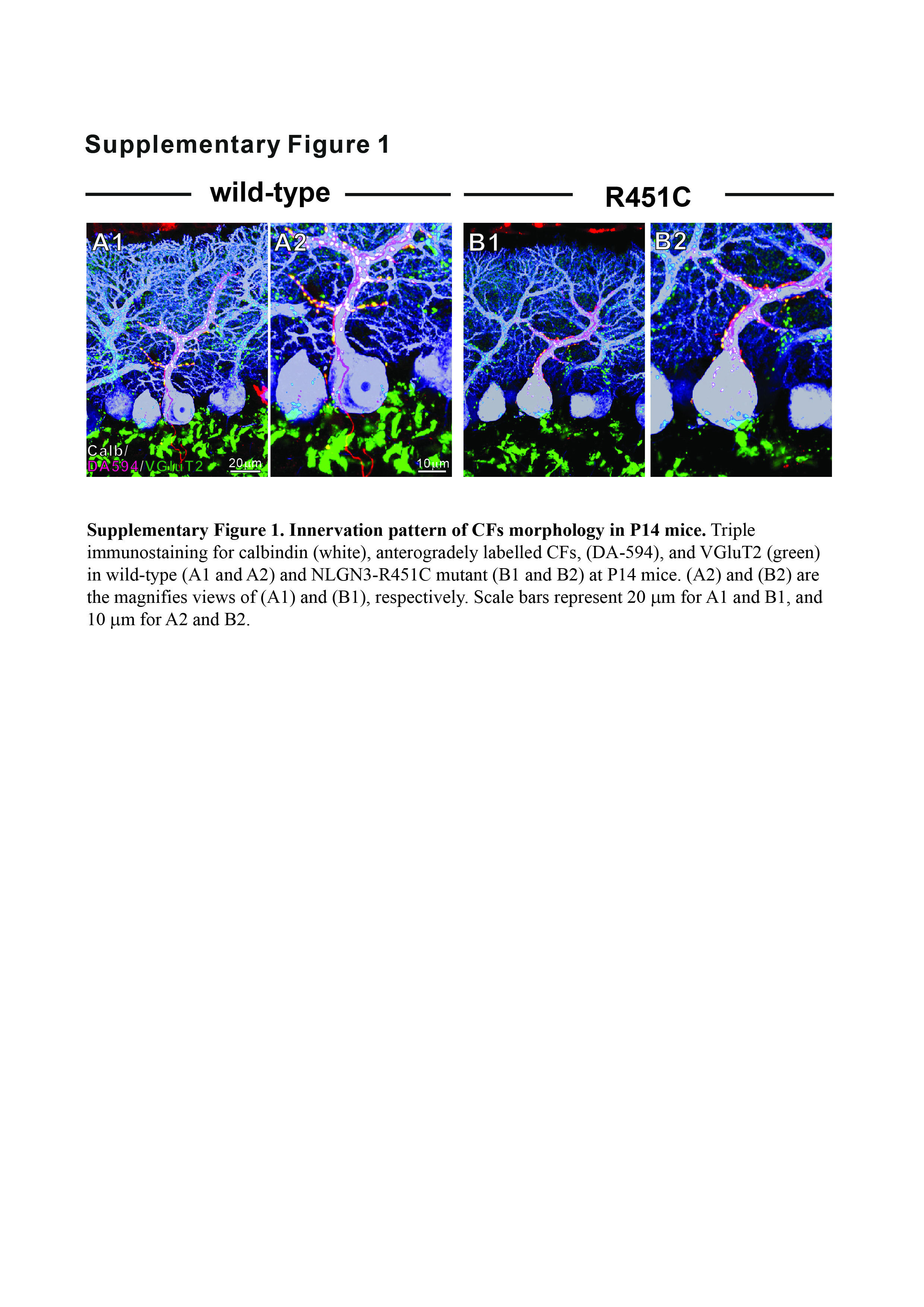

Supplement: Supplementary file 1 [file Image_1.JPEG]

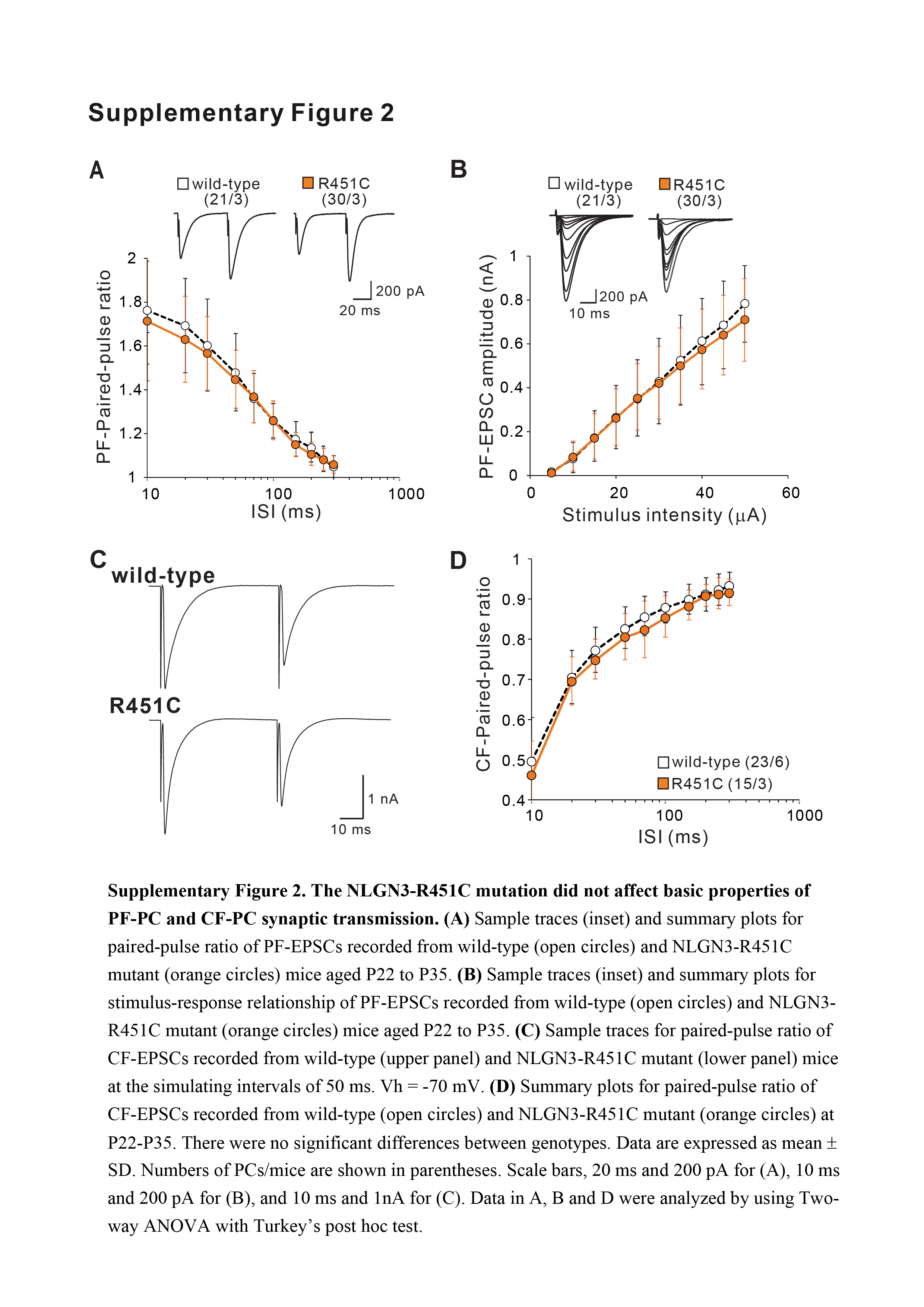

Supplement: Supplementary file 2 [file Image_2.JPEG]
